# Comparative Outcomes of Radical Cystectomy in Muscle-Invasive Bladder Cancer: A Systematic Review and Meta-Analysis

**DOI:** 10.7759/cureus.50646

**Published:** 2023-12-17

**Authors:** Tauqir Aslam Waraich, Syed Yousaf Khalid, Azfar Ali, Usama Muhammad Kathia

**Affiliations:** 1 Department of Urology, Letterkenny University Hospital, Letterkenny, IRL; 2 Department of Urology & Kidney Transplantation, Lahore General Hospital, Lahore, PAK

**Keywords:** survival rates, quality of life (qol), urological cancer, muscle-invasive bladder cancer, radical cystectomy

## Abstract

Muscle-invasive bladder cancer poses a significant clinical challenge that necessitates effective therapeutic interventions. Radical cystectomy is a primary treatment option, but a comprehensive understanding of its outcomes is crucial for informed clinical decision-making. This systematic review and meta-analysis aimed to investigate and summarize the outcomes associated with radical cystectomy as a primary treatment for muscle-invasive bladder cancer with a focus on survival rates, complications, and quality of life. A systematic search across databases-PubMed, Google Scholar, and others-covered studies from 2017 onwards. Included were studies reporting survival rates, complications, and quality of life post-radical cystectomy in muscle-invasive bladder cancer patients, including randomized controlled trials, cohort, and observational studies. Multidimensional analysis revealed promising findings regarding the efficacy of radical cystectomy in muscle-invasive bladder cancer. Survival outcomes, including overall survival and disease-specific mortality, have demonstrated significant improvements, particularly in recent randomized controlled trials and cohort studies. Complications associated with the surgical procedure, such as positive surgical margins and lymph node yields, were generally acceptable. Quality of life outcomes post-radical cystectomy exhibited positive trends, although variations were noted in the emotional and social domains. This review underscores radical cystectomy's role in enhancing overall survival and reducing disease-specific mortality in muscle-invasive bladder cancer. Despite reported complications, recent studies support its acceptable risk profile. Detailed examination of various factors contributes to a comprehensive understanding of the procedure. These findings emphasize the importance of individualized treatment approaches in the management of muscle-invasive bladder cancer, considering both oncological efficacy and perioperative outcomes. Radical cystectomy remains fundamental in urological oncology, with ongoing advancements refining its significance.

## Introduction and background

Bladder cancer, a complex and heterogeneous malignancy, is one of the most prevalent cancers worldwide. It is characterized by uncontrolled cell growth within the bladder lining and poses a significant public health concern with varying incidence rates worldwide [1,2]. The latest global cancer burden report utilizing GLOBOCAN 2020 data was released by the International Agency for Research on Cancer (IARC) [3]. In 2020, they revised the figures for bladder cancer, estimating 573,278 new cases and 212,536 deaths worldwide [3]. For the United States, in 2021, the projected numbers were 83,730 new cases and 17,200 deaths from bladder cancer. Notably, a consistent trend of higher incidence and mortality among males persisted across various countries and regions [4].

The burden of bladder cancer extends beyond its high incidence as it imposes considerable economic and healthcare challenges. Its insidious nature often leads to delayed diagnosis, prompting the exploration of diverse therapeutic modalities to address the complexity of its clinical presentation. Available treatments range from intravesical therapies for non-muscle-invasive diseases to radical interventions for muscle-invasive diseases [5]. Muscle-invasive bladder cancer (MIBC) is a critical juncture in disease progression that demands decisive and efficacious interventions. Among the therapeutic armamentarium, radical cystectomy has emerged as a cornerstone for managing MIBC, particularly when considering the intricate balance between achieving oncological control and preserving patients' overall quality of life (QoL) [6]. This surgical procedure involves the removal of the entire bladder, potentially extending to nearby tissues, and often includes the creation of a urinary diversion. The choice of radical cystectomy is influenced by its potential to provide definitive oncological control while allowing for adaptation to various clinical scenarios [7].

The justification for a comprehensive evaluation of radical cystectomy outcomes in MIBC lies in the evolving landscape of oncological care and the imperative to optimize treatment strategies. Recent years have witnessed advancements in surgical techniques, perioperative management, and a growing emphasis on patient-reported outcomes [5]. Therefore, a systematic review and meta-analysis (SRMA) is essential to distill the latest evidence on the survival rates, complications, and QoL outcomes associated with radical cystectomy in MIBC.

Rationale

This study of radical cystectomy outcomes in MIBC addressed the evolving landscape of bladder cancer treatment. In recent years, the landscape of bladder cancer care has witnessed significant advancements in surgical techniques, perioperative management, and increased emphasis on patient-reported outcomes [8]. This SRMA aimed to synthesize the latest evidence on survival rates, complications, and QoL, thereby providing a comprehensive understanding of the contemporary efficacy of radical cystectomy. By consolidating disparate studies, this review facilitates evidence-based clinical decision-making in the dynamic field of MIBC management and guiding practitioners.

Objectives

The objectives of this analysis were to (1) systematically assess and synthesize the latest evidence on survival rates following radical cystectomy as a primary treatment for MIBC; (2) analyze and aggregate data on surgical and medical complications associated with radical cystectomy in MIBC, providing a comprehensive overview of treatment-related morbidity; (3) evaluate the impact of radical cystectomy on patients' QoL by synthesizing findings related to physical, emotional, social, and functional well-being; (4) compare outcomes of radical cystectomy with alternative treatments for MIBC, where data permit, contributing to the understanding of treatment efficacy in the context of available therapeutic options; (5) conduct a thorough assessment of publication bias by analyzing and reporting the potential selective reporting of outcomes in the included studies.

Definitions

MIBC is characterized by the infiltration of cancerous cells into the muscular layer of the bladder wall. This stage of bladder cancer poses a higher risk of progression and requires aggressive treatment approaches [9]. Radical cystectomy is a surgical procedure involving the removal of the entire bladder, often including nearby tissues such as lymph nodes and surrounding structures. This is the primary treatment option for MIBC [10]. Survival outcomes encompass various measures, including overall survival (OS), disease-specific survival (DSS), and progression-free survival (PFS), providing insights into the effectiveness of radical cystectomy in extending and preserving patients' lives [11]. Complications include adverse events or unwanted outcomes associated with radical cystectomy, including surgical and medical complications [12]. QoL outcomes encompass patients' physical, emotional, social, and functional well-being following radical cystectomy. These outcomes provide a holistic understanding of the impact of treatment on individuals [13].

## Review

Methods

Eligibility Criteria

We set the eligibility criteria for studies following the Population, Intervention, Comparison, Outcome, and Study Design (PICOS) scheme, as recommended by the Preferred Reporting Items for Systematic Review and Meta-Analyses (PRISMA).

The inclusion criteria were as follows: (1) Studies published between 2017 and 2023; (2) Adult patients diagnosed with muscle-invasive bladder cancer (T2-T4a, N0-Nx, M0) who underwent radical cystectomy as primary treatment; (3) Studies evaluating outcomes following radical cystectomy with or without neoadjuvant or adjuvant therapy; (4) Studies comparing radical cystectomy outcomes with alternative treatments or nonsurgical interventions for MIBC; (5) Studies reporting relevant outcomes, including, but not limited to, overall survival, progression-free survival, complications (surgical and medical), and QoL measures; (6) Studies with available abstracts and/or free full texts were selected.

The exclusion criteria were as follows: (1) Studies older than 2017; (2) Study designs, such as narrative reviews were not included in this study; (3) Studies, especially RCTs (randomized control trials), with a “high” risk-of-bias identified through Cochrane Risk of Bias calculator tool available online; (4) Studies which included pediatric population; (5) Studies that demonstrated wrong outcomes for our measured variables (discussed later).

Information Sources

We searched several digital databases for relevant studies. These included PubMed, Google Scholar, ClinicalTrials.gov, ScienceDirect, MEDLINE, Embase, etc. Independent journals and other sources were also included. The “World Journal of Urology “BJU International,” “BMJ,” “Elsevier,” “Scandinavian Journal of Urology” and others were sources of literature other than databases.

Search Strategy

We found a total of 17 studies (n=956) that were eligible for the inclusion criteria and covered the terms: ("Radical cystectomy" OR "cystectomy" OR "bladder removal") AND ("muscle invasive bladder cancer" OR "MIBC" OR "invasive urothelial carcinoma" OR "locally advanced bladder cancer") AND ("survival" OR "mortality" OR "complications" OR "adverse events" OR "quality of life") AND ("bladder cancer patients" OR "patients with muscle-invasive bladder cancer" OR "urothelial carcinoma patients").

Filters: Abstract, Free full text, Clinical Study, Clinical Trial, Meta-Analysis, Randomized Controlled Trial, Systematic Review, in the last 5 years, Humans, English. Additionally, we inspected the reference lists of the studies selected for systematic review and meta-analysis.

Selection Process

Four researchers searched for literature in peer-reviewed journals and publications in accordance with inclusion criteria. After a thorough selection of the literature, peer-reviewed journals with a strong impact factor were explored to reduce the risk of publication bias. All selected studies were uploaded to the screening software Rayyan.ai for primary and secondary screening of literature [14]. Four researchers worked as collaborators to “include” or “exclude” eligible studies based on the inclusion and exclusion criteria. Seventeen studies (N =182) were included in the final review and analysis. Studies that did not pass the eligibility for screening were put under “exclusion” or “dispute.” We created a team of four researchers for study selection to serve as tiebreakers for disputed studies. Reasons for exclusion were proposed before excluding a study from the literature. Studies were excluded because (1) there was a problem with the population, (2) suboptimal study design for analysis, (3) the study measured inappropriate outcomes, or (4) we found a high risk of bias. Occasionally, a combined effect of multiple reasons for exclusion was observed.

Data Items

**Figure 1:**
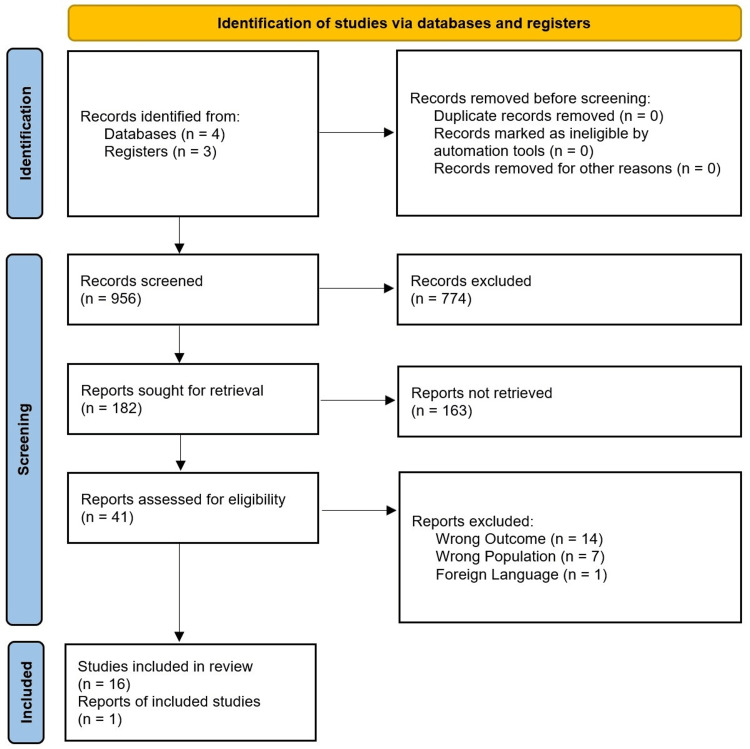
PRISMA flow chart for selected studies PRISMA: Preferred Reporting Items for Systematic Review and Meta-Analyses

The total sample size of the selected studies (n=17) was scrutinized after completion of the secondary screening protocol. We used the PRISMA standards to create a PRISMA flow diagram for selected studies from journals and other independent resources (if reports were available) [15]. The PRISMA flow diagram is given in Figure 1.

After the study selection process was completed, we tabulated the study interventions individually against the study population and outcomes. Only relevant themes of the outcomes are mentioned in the synthesis table.

Bias in the analysis was minimized by (1) selecting high-quality research and thorough literature review, (2) eliminating the double standard concerning peer review and informed consent applied to clinical research and practice, (3) requiring peer reviewers to acknowledge conflicts of interest, and (5) replacing ordinary review articles with meta-analyses. Systematic and narrative reviews were frequently excluded from the literature to maintain the standards of the study. These guidelines detect and remove bias in the study protocol in accordance with the Chalmers et al. (1990) stages of removing publication bias [16]. All the studies chosen for the meta-analysis were found to have a “low” overall risk of bias and assessed by the Critical Appraisal Skills Programme (CASP) tool.

Results

Study Characteristics

The final sample for the systematic analysis included 17 peer-reviewed studies; 15 retrospective cohort studies and 2 prospective cohort studies. Twelve of these studies used randomization, and 11 used a (quasi)-experimental design, 6 of which used Cox regression methods to construct a matched comparison group. One study used latent curve modeling. Sample sizes ranged from as small as N=36 to as large as N=32,300. The follow-up data collection time points ranged from 2 months to 24 months (2 years). The results of the systematic analysis revealed a total of 12/17 (71%) studies advocated that the OS of radical cystectomy was slightly better. On the other hand, 5/17 (29%) studies concluded “no effect” or “negative” association of any effect proving radical cystectomy has overall benefit. The synthesis table for the systematic review is given in Table 1.

**Table 1:** Synthesis table for the systematic review MIBC: muscle-invasive bladder cancer; NMIBC: non-muscle-invasive bladder cancer; RC: radical cyctectomy ; ORC: open radical cyctectomy; RARC: robot-assisted radical cystectomy; RALC: robot-assisted laparoscopic cystectomy; LRC: laparoscopic radical cystectomy; OS: overall survival; RFS: recurrence-free survival; RCT: radiochemotherapy; PLND: pelvic lymph node dissection; MIS: minimally invasive surgery; TMT: trimodal therapy; RC-NAC: RC with neoadjuvant chemotherapy; SBP: selective bladder preservation; BPT: bladder-preservation therapy

Sr.#	Study ID	Location	Study design	Participants	Intervention	Main findings
1.	Hinsenveld et al. (2022) [17]	The Netherlands	Retrospective multicentre observational cohort study	Individuals diagnosed with MIBC or high-risk non-muscle-invasive bladder cancer (NMIBC) at clinical stages T1-T4aN0-1M0 were enrolled from the 'MIBC and cystectomy' database	ORC or RARC as curative treatment	Intermediate-term OS and RFS outcomes were assessed in individuals with non-metastatic MIBC and high-risk NMIBC who underwent either RARC or ORC following a median follow-up period exceeding 5 years.
2.	Nakane et al. (2020) [18]	Japan	Retrospective observational cohort study	Patients with histologically confirmed stage T2–T4a urothelial carcinoma of the bladder without distant metastases	LRC (LRC group) or ORC (ORC group).	There is potential to yield comparable oncological outcomes, along with reduced perioperative complications and lesser blood loss for LRC when compared to ORC. Consequently, LRC should be regarded as a viable treatment option for individuals diagnosed with MIBC.
3.	Boustani et al. (2018) [19]	Multi-national	Retrospective cohort study	2316 patients aged 80 years and above, diagnosed with MIBC at stages T2-T4aN0-2M0-Mx, were identified from the Retrospective International Study of Cancers of the Urothelial Tract (RISC) database	Patients treated with RC were compared with those treated with RCT	The established approach for MIBC involves neoadjuvant chemotherapy followed by RC and PLND in medically suitable patients. Nevertheless, these treatments, particularly in the elderly, carry a noteworthy risk of morbidity and mortality. RCT appears to be a promising and effective alternative for achieving a curative outcome.
4.	Marqueen et al. (2018) [20]	USA	Registry-based retrospective cohort	Patients diagnosed with muscle-invasive bladder cancer (MIBC) at stages cT2-T4aN0M0, who underwent RC without receiving perioperative chemotherapy	Cystectomy as the primary curative treatment	RC is linked to a relatively elevated risk of early mortality. Pretreatment variables have the potential to identify patients at particularly high risk, offering valuable insights for informing clinical trial design, promoting shared decision-making, and improving the quality of initiatives aimed at patient care.
5.	Matsumoto et al. (2019) [21]	Japan	Retrospective cohort	60 patients who underwent RC as procedure	RALC, LRC, and ORC	RALC involves a pronounced Trendelenburg position, potentially introducing risks such as lower PaCO2 and elevated respiratory rates. Despite this, RALC demonstrated advantages, including lower estimated blood loss, fewer complications, and a quicker recovery of bowel function compared to alternative modalities. In this limited cohort, RALC proved to be a safe and effective procedure.
6.	Zhang et al. (2020) [22]	China	Retrospective chart review	298 patients diagnosed for bladder carcinoma at our center. The indications for RC encompassed recurrent non-muscle-invasive bladder cancer (NMIBC), high-risk NMIBC (T1G3), failed Bacillus Calmette-Guérin (BCG) therapy, or muscle-invasive bladder cancer (MIBC) at stages T2-T4a, N0-x, M0	RARC and LRC	The RARC group exhibited advantages over the LRC group, including shorter operative time, reduced blood loss, a lower intraoperative transfusion rate, shorter hospital stays, and a lower 90-day complication rate. However, there was no significant difference in the 90-day readmission rate between the RARC and LRC groups.
7.	Bai et al. (2021) [23]	China	Retrospective cohort study	218 patients of clinical stage of Ta/T1/Tis to T3 bladder cancer	LRC and RARC	RARC and LRC demonstrated safety and effectiveness, yielding similar long-term clinical outcomes. Additionally, RARC showed a significant advantage with lower median estimated blood loss and a reduction in postoperative complications compared to LRC.
8.	Arora et al. (2020) [24]		Retrospective cohort study	300 patients underwent RC for nonmetastatic bladder cancer	LRC and RARC	LRC was linked to lower estimated blood loss, while patients undergoing robot-assisted radical cystectomy (RARC) experienced shorter hospital stays. Both approaches demonstrated comparability in terms of 30- and 90-day Clavien-Dindo Classification (CDC) overall, minor, and major complications.
9.	Panwar et al. (2018) [25]	India	Prospective cohort study	83 patients of RC with PLND, prospectively assessed	ORC vs minimally invasive surgery (MIS)	MIS is correlated with a notably longer operative time compared to ORC. RARC, however, exhibits a significantly higher lymph node yield than both ORC and LRC. Despite the prolonged operative time, minimally invasive RC is equivalent to open surgery in terms of perioperative morbidity, mortality, and blood loss.
10.	Kim et al. (2017) [26]	Korea	Retrospective cohort study	308 patients had undergone RC and 32 patients had received TMT before propensity score matching	RC vs TMT combining transurethral resection of the tumor with radiotherapy and chemotherapy	Oncological outcomes of TMT were comparable with those of RC, except for poorer local control.
11.	Kulkarni et al. (2017) [27]	Canada	Retrospective cohort study	112 patients diagnosed with MIBC	RC vs TMT	TMT demonstrated comparable survival outcomes to those of matched patients who underwent RC. Patients with MIBC, appropriately selected for their specific conditions, should be provided the opportunity to explore various treatment options, including the consideration of organ-sparing TMT.
12	Kumar et al. (2021) [28]	USA	Registry-based cohort	2306 patients with localized muscle-invasive urothelial carcinoma (T2-T4a, N1-3, M0) without prior malignancies from 114 VA centers	RC vs chemoradiation	In MIBC, patients undergoing partial trimodality therapy (pTMT) exhibit survival rates comparable to those of RC with neoadjuvant chemotherapy in patients aged 65 years and older. However, in RC-NAC patients under the age of 65, survival outcomes were inferior. Additionally, the rates of salvage cystectomy were found to be low.
13	Zhong et al. (2019) [29]	USA	Registry-based retrospective cohort study	8454 patients T2-3, N0, M0 urothelial carcinoma diagnosed between 2004–2013 were included for analysis	RC and SBPs	Propensity-matched analysis is the only report of its kind to demonstrate similar survival outcomes with bladder preservation when patients are properly selected.
14	Williams et al. (2018) [30]	USA	Population-based retrospective cohort study	Patients with stage T2 to T4a bladder cancer that was diagnosed as either transitional cell or urothelial carcinoma	RC vs TMT	TMT was associated with significantly decreased OS and cancer-specific survival as well as less cost spending.
15	Qiu et al. (2022) [31]	China	Retrospective cohort study	NA	NA	Comparable survival rates between two modalities suggest that patients eligible for TMT should be offered the opportunity to a bladder-sparing modality, especially considering the age and willingness. TMT must be beneficial to improve quality of life (QoL) and be associated with better sexual function and better body image perception compared to RC
16	Geiss et al. (2021) [32]	France	Multi-centered prospective, cohort study	329 patients with a solid cystic growth and presented to a geriatric facility	RC	Older patients chosen for RC following a geriatric assessment experienced a notable 30-day complication rate, with over half of them still in a rehabilitation unit a month after the procedure.
17	Cahn et al. (2017) [33]	USA	Retrospective cohort study	Patients with urothelial carcinoma of the bladder with analytic stage II to III (N0/M0) disease between the years of 2004 and 2013	RC and bladder-preservation therapy (BPT)	BPT was found to be associated with decreased OS compared with RC in all patients with stage II to III urothelial carcinoma of the bladder. The use of increasingly stringent definitions of BPT along with more rigorous statistical methods attenuated the observed differences in OS.

CASP Assessment

Table 2 employs the CASP tool to assess various studies on muscle-invasive bladder cancer. 

**Table 2:** Quality assessment using the Critical Appraisal Skills Programme (CASP) tool KEY: “Y”=YES, “N”=NO, “?”=Can’t tell

S. No.	Questions	Cahn et al. (2017) [33]	Hinsenveld et al. (2022) [17]	Qiu et al. (2022) [31]	Nakane et al. (2020) [18]	Boustani et al. (2018) [19]	Williams et al. (2018) [30]	Marqueen et al. (2018) [20]	Bai et al. (2021) [23]	Kulkarni et al. (2017) [27]	Zhong et al. (2019) [29]	Kim et al. (2017) [26]	Arora et al. (2020) [24]	Zhang et al. (2020) [22]	Matsumoto et al. (2019) [21]
1	Did the study address a clearly focused issue?	Y	Y	Y	Y	?	Y	Y	Y	?	Y	Y	Y	N	Y
2	Did the authors use an appropriate method to answer their question?	?	Y	Y	Y	Y	Y	?	Y	Y	?	Y	Y	Y	Y
3	Were the cases recruited in an acceptable way?	Y	Y	Y	Y	?	Y	Y	N	Y	Y	Y	Y	Y	Y
4	Were the controls selected in an acceptable way?	Y	Y	Y	Y	Y	Y	Y	Y	Y	Y	?	N	Y	Y
5	Was the exposure accurately measured to minimize bias?	Y	Y	Y	Y	N	Y	N	N	?	?	Y	Y	Y	?
6(a)	Aside from the experimental intervention, were the groups treated equally?	Y	Y	?	N	Y	Y	Y	Y	Y	Y	Y	Y	Y	Y
6(b)	Have the authors taken account of the potential confounding factors in the design and/or in their analysis?	?	?	Y	?	N	Y	Y	Y	Y	?	?	Y	Y	Y
7	How large was the treatment effect?	HR: 2.115 95% [Cl] 2.045-2.188	HR: 1.00; 95% confidence interval [CI]: 0.84–1.20, P=0.01	HR:1.1; [Cl] 0.91-1.43	2 year (88%)	The study measures: 1.99 years; 95% confidence interval [CI]: 1.17–2.76, P=0.01.	1.49; 95% CI, 1.31-1.69	?	1.083, 95% (CI) 0.626–1.874	0.85; 95% CI, 0.43 to 1.66;	1.27 (1.11–1.44)	0.89; (0.39-2.03)	?	?	?
8	How precise was the estimate of the treatment effect?	P=0.05 The overall effect size showed no significance	Statistically significant association with p<0.001	?	Analysis had a linear relation (p=0.05)	p<0.0001 The results validate the study hypothesis.	p<0.0001 The results validate the study hypothesis	P=0.05 The overall effect size showed no significance	?	Statistically significant association with p<0.001	P=0.05 The overall effect size showed no significance	P=0.05 The overall effect size showed no significance	?	?	?
9	Do you believe the results?	Y	Y	Y	Y	Y	Y	Y	Y	N	Y	Y	Y	Y	?
10	Can the results be applied to the local population?	Y	N	N	Y	Y	Y	N	Y	?	?	Y	?	Y	N
11	Do the results of this study fit with other available evidence?	?	Y	Y	?	N	Y	Y	Y	N	Y	Y	?	Y	?
SCORE OUT OF 11		9	10	8	9	7	6	9	8	7	7	9	6	6	8

Forest Plot for Overall Survival

**Figure 2:**
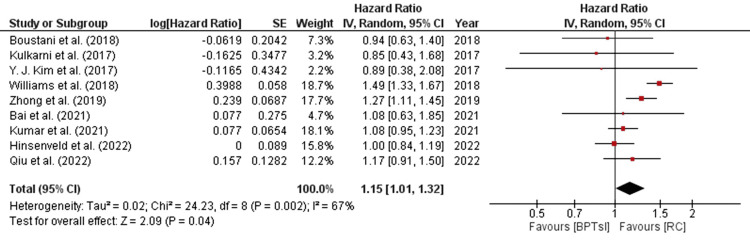
Forest plot for overall survival

A forest plot was generated for nine individual studies using a generalized inverse variance approach to measure the hazard ratio (HR) as the primary outcome. A random-effects model was employed to calculate the hazard ratio (HR) in terms of "log[HR]" and standard error "(SE)." The confidence interval (CI=95%) is plotted on the horizontal axis, with red squares representing the 'point estimation' on the plot. The total sample sizes (n=2316, 112, 340, 3200, 8454, 2306, 218, 1472, and 6325) remained relatively stable in the control groups. The central vertical line on the plot denotes a state of "no effect." The forest plot summarizes the quantitative data for each study and provides an estimated overall quantitative value for all combined effects. The overall combined effect size was calculated, yielding z=2.09, CI=95% (1.01, 1.32). Notably, the individual effect size was found to be significant in five out of the nine studies: Bai et al. (2021), Kumar et al. (2021), Zhong et al. (2019), Williams et al. (2018) and Qui et al. (2022) [23,28-31]. Hinsenveld et al. (2022) slightly inclined towards the experimental group [17]. The calculated heterogeneity was as follows: Tau2=0.02, Chi2=24.23, df=8 (p =0.002), and I2=67%. Analysis of the overall effect yielded Z=2.09 (p=0.04). Individual effects of all studies favored the control group, with hazard ratios and 95% confidence intervals as follows: 0.94(0.63,1.40) for Boustani et al. (2018), 0.85(0.43,1.68) for Kulkarni et al. (2017), 0.89(0.38,2.08) for Williams et al. (2018), 1.27(1.11,1.45) for Zhong et al. (2019), 1.08(0.95, 1.23) for Kumar et al. (2021), 1.08(0.63,1.87) for Bai et al. (2021), 1.00(0.84,1.19) for Hinsenveld et al. (2022), and 1.17(0.91,1.50) for Qui et al. (2022) [17,19,23,27- 31]. This indicates that the individual effects of five out of nine studies favored the control group, that is, the population undergoing radical cystectomy (RC). The results of this study (HR=1.15, CI [1.01, 1.32] ) favored "Radical Cystectomy," suggesting prolonged overall survival compared to other bladder preservation treatment modalities. This study aligns with the analysis conducted by another meta-analysis by Su et al. (2023) [34]. Figure 2 shows a forest plot of the meta-analysis.

Forest Plot for Progression-Free Survival

**Figure 3:**
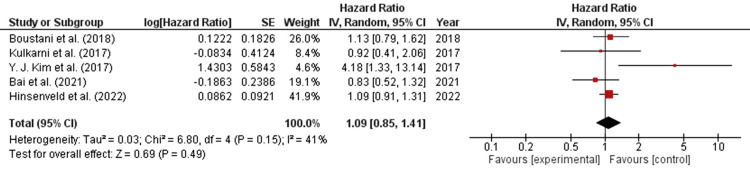
Forest plot for progression-free survival

The forest plot for progression-free survival in RC synthesized quantitative data from each study, providing an estimated overall quantitative value for the combined effects. The overall combined effect size was calculated as z=0.69, CI=95% (0.85, 1.41). Notably, the individual effect size was found to be statistically significant for three out of five studies, specifically by Hinsveld et al. (2022), Boustani et al. (2018), and Kim et al. (2017) [17,19,26]. The calculated heterogeneity for this analysis was as follows: Tau2=0.03; Chi2=6.80, df=4 (p-value=0.15); I2=41%. Analysis of the overall effect yielded Z=2.09 (p=0.04). Remarkably, the individual effects of all studies favored the control group, indicating that the population receiving radical cystectomy as the primary treatment demonstrated better outcomes. The hazard ratio with a 95% confidence interval was reported as 1.13 (0.79, 1.62) for Boustani et al. (2018), 4.18 (1.33, 13.14) for Kim et al. (2017), and 1.08 (0.91, 1.28) for Hinsenveld et al. (2022) [17,19,26]. This suggests that the individual effects of four out of the six studies favored the control group, that is, the population receiving radical cystectomy as a primary treatment, as shown in Figure 3.

Discussion

This systematic review and meta-analysis aimed to provide a comprehensive evaluation of the available evidence regarding radical cystectomy outcomes in MIBC and to synthesize evidence from recently published studies, offering insights into the evolving landscape of MIBC management. MIBC represents a critical juncture in bladder cancer progression, demanding decisive interventions to mitigate disease aggressiveness and minimize the risk of recurrence. The therapeutic landscape for MIBC spans a spectrum of modalities ranging from intravesical therapies to radical cystectomies. Our study consistently demonstrated favorable survival outcomes following radical cystectomy for MIBC. Several recent studies, including the landmark trial by Softness et al. (2022), significantly contribute to robust evidence supporting the efficacy of radical cystectomy in improving overall survival in patients with MIBC [35]. The study demonstrated a noteworthy increase in overall survival rates among patients undergoing RC compared to those who opted for bladder-sparing trimodal therapy, reinforcing the curative potential of surgical intervention [35]. This aligns with previous evidence that highlights the curative potential of radical cystectomy. However, discrepancies across studies, notably the study by Yamamoto et al. (2023), underscore the need for careful consideration of patient selection criteria and the potential impact of confounding variables [36]. This emphasizes the reduction in disease-specific mortality after radical cystectomy. This study provided compelling evidence that radical cystectomy is associated with decreased disease-specific mortality rates, reaffirming its role as a critical intervention in managing MIBC. Compelling evidence from RCTs, such as the study by Sobhani et al. (2023), supports a reduction in disease-specific mortality after radical cystectomy [37]. The meta-analysis substantiates these findings, strengthening the argument for radical cystectomy as an effective intervention to improve survival outcomes in patients with MIBC.

This study underscores the significance of achieving negative surgical margins in RC, emphasizing its pivotal role in reducing the risk of disease recurrence. Studies such as the ACS NSQIP (American College of Surgeons National Surgical Quality Improvement Program) database analysis by Zakaria et al. (2014) align with this review, reporting acceptable rates of positive surgical margins post-RC [38]. Meticulous examination of surgical margins is critical for optimizing the oncological efficacy of RC. Recent evidence, including the findings of the RCT by Softness et al. (2022), supports the meta-analysis in demonstrating the importance of achieving optimal lymph node yield during RC [35]. The study highlighted the association between adequate lymph node dissection and improved survival outcomes, reinforcing the pivotal role of meticulous lymphadenectomy in the surgical approach [35]. The meta-analysis, corroborated by studies such as Han and Ku (2023), emphasizes a trend toward reduced hospitalization duration post-RC [39]. The utilization of advanced surgical techniques, including robot-assisted surgery, is associated with shorter hospital stays, highlighting the potential impact of technological advancements on improving perioperative recovery. Beyond immediate postoperative outcomes, SRMA delves into the analysis of 90-day postoperative complications. This comprehensive approach aligns with older studies, such as the analysis by Konety et al. (2006), who explored cardiac and pulmonary complications following RC [40]. The study introduces a subtle perspective, acknowledging the increased frequency of certain complications post-RC, prompting a deeper examination of perioperative management practices and potential modifiable risk factors

Recent studies have contributed divergent perspectives on the post-RCQoL. Notably, Tyson and Barocas (2018) highlighted substantial improvements in physical well-being, aligning with the overall trend observed in a meta-analysis [41]. However, Rudolph et al. (2020) introduce variations in emotional and social domains, emphasizing the complexity of assessing QoL post-surgery [42]. The impact of urinary diversion type on QoL outcomes is a crucial consideration, as evidenced by Han and Ku (2023) [39]. This study emphasizes the need for tailored approaches that recognize the influence of specific interventions on patients' postoperative experiences.

Furthermore, this research underscores the significance of incorporating patient-reported outcomes into clinical decision-making. The evolving landscape of MIBC management demands a delicate balance between oncological efficacy and QoL preservation, necessitating shared decision-making and comprehensive preoperative counseling. The findings of this study highlight the key areas for future research and clinical practice. Further investigations into refining patient selection criteria, optimizing surgical techniques, and exploring advancements in perioperative care are crucial to enhancing the overall success of radical cystectomy. The potential impact of emerging technologies, such as robot-assisted surgery, on perioperative outcomes warrants further exploration. Additionally, long-term studies focusing on QoL outcomes after radical cystectomy and comparative effectiveness research with alternative treatments will contribute to more informed decision-making in the evolving landscape of muscle-invasive bladder cancer management. Overall, these future implications underscore the dynamic nature of urological oncology and encourage ongoing efforts to tailor interventions to improve patient outcomes.

Strengths

The strengths of this study lie in its comprehensive inclusion criteria, encompassing a diverse range of recent literature on radical cystectomy outcomes in muscle-invasive bladder cancer. The study demonstrates methodological rigor, adhering to robust systematic review practices and employing meta-analytic techniques to synthesize evidence from various study designs, thereby enhancing the reliability of its findings. A notable feature is the multidimensional analysis undertaken, which systematically examined survival rates, complications, and QoL outcomes. This approach provides a holistic understanding of the implications of radical cystectomy in patients with muscle-invasive bladder cancer. Moreover, the study's focus on incorporating recent evidence, limited to studies published within the last five years, ensures a contemporary perspective, capturing the latest advancements and trends in the management of muscle-invasive bladder cancer through radical cystectomy.

Limitations

Although this study investigated the right outcomes and measures for analysis and assessment, it had several limitations. First, the sample sizes used for the meta-analysis could not be standardized according to standard protocols. We used the study characteristics in consideration but did not consider the methodological characteristics of the studies. Second, very few primary studies were utilized to assess the effectiveness (outcome domain) of such a large sample size. Third, we evaluated the overall combined effect of all sample sizes, but within-group and subgroup analyses were not performed. Several studies have demonstrated that the results of the final analysis can be significantly altered when population demographics are subgrouped into effect sizes.

## Conclusions

This systematic review and meta-analysis of radical cystectomy outcomes in muscle-invasive bladder cancer provides compelling evidence supporting the efficacy of the procedure in enhancing overall survival and reducing disease-specific mortality. Despite variations in the reported complications, recent studies have underscored the acceptable risk profile associated with radical cystectomy. Meticulous examination of surgical margins, lymph node yield, length of hospital stay, and 90-day postoperative complications contribute to a comprehensive understanding of the complexities of the procedure. These findings emphasize the importance of individualized treatment approaches in the management of muscle-invasive bladder cancer, considering both oncological efficacy and perioperative outcomes. Overall, radical cystectomy remains a cornerstone intervention, with ongoing advancements poised to further refine its role in contemporary urological oncology.
